# Immune effects of lipids isolated from *Oncorhynchus mykiss* eggs on cyclophosphamide-induced immunosuppression in mice

**DOI:** 10.3389/fnut.2026.1842601

**Published:** 2026-09-03

**Authors:** One Jun Kim, JeongUn Choi, Weerawan Rod-In, A-Yeong Jang, Purna Chandra Mashurabad, Sang-Min Lee, Seok Kyu Jung, Woo Jung Park

**Affiliations:** 1 Department of Wellness-Bio Industry, Kangwon National University, Gangneung, Gangwon, Republic of Korea; 2 Department of Marine Food Science and Technology, Kangwon National University, Gangneung, Gangwon, Republic of Korea; 3 Department of Agricultural Science, Faculty of Agriculture, Natural Resources and Environment, Naresuan University, Phitsanulok, Thailand; 4 Center of Excellence in Research for Agricultural Biotechnology, Naresuan University, Phitsanulok, Thailand; 5 East Coast Life Science Institute, Kangwon National University, Gangneung, Gangwon, Republic of Korea; 6 Department of Aquatic Life Medicine, Kangwon National University, Gangneung, Gangwon, Republic of Korea; 7 Department of Horticultural Science, Kongju National University, Yesan-gun, Chungcheongnam-do, Republic of Korea; 8 Department of Marine Convergence Science, Kangwon National University, Gangneung, Gangwon, Republic of Korea; 9 KBIoRANCh Co., Ltd, Gangneung, Gangwon, Republic of Korea

**Keywords:** cyclophosphamide, immunomodulatory, lipids, PEG, rainbow trout

## Abstract

Rainbow trout (*Oncorhynchus mykiss*) eggs are a rich source of polyunsaturated fatty acids (PUFAs), with levels exceeding those found in fish meat, and are also a notable source of bioactive omega-3 fatty acids, primarily eicosapentaenoic acid (EPA)and docosahexaenoic acid (DHA). However, despite their nutritional advantages, their *in vivo* immunomodulatory properties remain poorly understood. This study explored the immunomodulatory potential of the lipid extract from *O. mykiss* eggs combined with PEG6000 (OM-PEG) in a cyclophosphamide (CY)-induced immunosuppressive BALB/c mouse model. Mice were randomly assigned to nine groups (*n* = 3/group): normal control, CY, CY + ginseng (100 mg/kg), CY + levamisole (40 mg/kg), CY + PEG6000 (10 mg/kg), and four CY + OM-PEG groups (50–200 mg/kg). In CY-treated mice, prophylactic oral administration of OM-PEG (50-200 mg/kg body weight) protected against changes in spleen size and index. It also significantly stimulated splenic immune cells, including T cells, B cells, and natural killer (NK) cells (*p* < 0.05). Furthermore, cell proliferation, nitric oxide (NO) production, and phagocytic activity were enhanced in peritoneal macrophages (*p* < 0.05). The mRNA expression of immunostimulatory cytokines and mediators, including interleukin-1β (IL-1β), IL-2, IL-4, IL-6, inducible nitric oxide synthase (iNOS), TNF-α, IFN-γ, and COX-2, was significantly upregulated in both splenic lymphocytes and peritoneal macrophages of CY-treated mice (*p* < 0.05). Consequently, these results suggest that OM-PEG6000, prepared by incorporating lipids isolated from *O. mykiss* eggs into PEG6000, contributes to immune system activation and has potential as a functional food ingredient due to its immune-enhancing efficacy.

## 1 Introduction

The immune system is a crucial defense mechanism that protects the body against tissue damage, infections, pathogens, and abnormal cells, thereby maintaining overall health (1). It is primarily composed of immune organs, immune cells, and immune molecules. Cyclophosphamide (CY) is a widely used chemotherapeutic agent that induces immunosuppression, myelosuppression, and cytotoxic effects, and its long-term use may damage normal cells (2, 3). CY can alter DNA structure, inhibit cell replication, and ultimately induce cell death (4). Several natural substances have been identified as effective immunomodulatory agents that may help mitigate the harmful side effects of chemotherapy and have demonstrated the ability to enhance immune function in mouse models of CY-induced immunosuppression (5–7).

Lipids consist mainly of fatty acids, which serve as a source of metabolic energy, while essential fatty acids perform specific physiological functions in the body (8). Lipids isolated from natural sources exhibit various biological activities, such as antibacterial, antiviral, antifungal, anticancer, antidiabetic, anti-inflammatory, and immunomodulatory effects (9–12). Recently, lipid extracts from *Arctoscopus japonicus* eggs were shown to alleviate CY-induced immunosuppression in BALB/c mouse models through various immunomodulatory activities, including enhancing the spleen index, improving NK cell cytotoxic activity and lymphocyte proliferation, stimulating immune factor secretion in splenic lymphocytes, and activating peritoneal macrophages (13).

*Oncorhynchus mykiss*, also known as rainbow trout, is a species native to North America, widely farmed and popular in both recreational and commercial fishing. It is a cold-water species that thrives in freshwater environments but can also be found in some saltwater habitats. The roe (eggs) of *O. mykiss* are commonly used in both research and culinary applications and are popular for their desirable taste and high nutritional value. *O. mykiss* eggs contain higher levels of polyunsaturated fatty acids (PUFAs) compared to fish meat, meeting recommended values for the PUFA/SFA ratio and the n-3/n-6 ratio, and providing significant amounts of eicosapentaenoic acid (EPA) and docosahexaenoic acid (DHA) (14). However, *O. mykiss* has generally been used as an animal model for testing various compounds and evaluating its immune function (15–17). Few studies have investigated natural compounds isolated from *O. mykiss*, even though these compounds have been shown to exhibit antimicrobial (18), antioxidant (19–21), anticancer (19, 20), and anti-obesity effects (22). Recently, *O. mykiss* lipids have been reported to exert immune effects by protecting immune cells (23). These studies demonstrated that *O. mykiss* exerts anti-inflammatiory effects through various physiological activities on RAW264.7 cells, and its primary chemical composition has been characterized. However, the immune-enhancing activity of lipids isolated from *O. mykiss* eggs has not yet been investigated. Therefore, this study evaluated the *in vivo* immunomodulatory effects of lipids isolated from *O. mykiss* eggs, and incorporated into PEG6000 (OM-PEG) in CY-induced immunosuppressed mice, highlighting its potential as a functional food ingredient.

## 2 Materials and methods

### 2.1 Materials

Red ginseng (Korea Ginseng Corp., Daejeon, Republic of Korea), levamisole and PEG6000 (Sigma-Aldrich, St. Louis, MO, USA), PBS (Biosesang, Yongin, Republic of Korea), RPMI 1640 (Gibco™, NY, USA), penicillin–streptomycin (WELGENE Inc., Gyeongsan, Republic of Korea), LPS (Sigma-Aldrich, St. Louis, MO, USA), Concanavalin A (Con A) (Sigma-Aldrich, St. Louis, MO, USA) and Neutral Red Solution (Sigma-Aldrich, St. Louis, MO, USA) were used in this study.

### 2.2 Samples

O. mykiss were provided by BNF Solution Co., Ltd. (Pyeongchang Trout Farm, Republic of Korea). They have been grown for approximately 22–24 months after hatching to develop eggs. The fish were euthanized, and their eggs were collected. The collected eggs were freeze-dried, and the resulting powder was immediately stored at −20 °C.

### 2.3 Preparation of OM-PEG

OM-PEG was prepared by incorporating lipid extract from *O. mykiss* eggs with PEG6000 as previously described (13). PEG6000 (polyethylene glycol), conjugated with lipids from *O. mykiss* eggs, is commonly used as an inert compound and a carrier for drug delivery (24). Lipids from the powdered eggs were extracted using a mixture of chloroform and methanol, following a modified Bligh and Dyer method (25). The lipid extract was then mixed with PEG6000 in a 1:1 ratio (w/w) at 60 °C, concentrated using a rotary evaporator, and stored at –20 °C. Finally, the OM-PEG was kept in a desiccator until further use. The OM-PEG was quantified by gas chromatography–flame ionization detection (GC-FID) following conversion to fatty acid methyl esters (FAMEs), as previously described (26).

### 2.4 Animal experimental design

6-week-old male BALB/c mice (21–23 g) were obtained from Koatech Animal Inc. (Pyeongtaek, Republic of Korea). The animals were housed under standard conditions, with the temperature maintained at 22 ± 2 °C and a 12-h dark/light cycle. The mice were fed a standard RodFeed diet (Hana Biotech, Pyeongtaek, Republic of Korea) and had *ad libitum* access to food and water throughout the experimental period. The RodFeed diet contained 20.0% protein, 3.54% fat, 4.50% fiber, 6.96% ash, 0.90% calcium, 0.72% phosphorus, and essential vitamins and amino acids. All experimental procedures were approved by the Institutional Animal Care and Use Committee (IACUC) of Gangneung–Wonju National University, Korea (approval number: GWNU-2021-12).

After one week of acclimation, Mice were randomly assigned to nine experimental groups, with three mice per group. The experimental groups were as follows:

Group 1: Normal control group (NC; physiological saline only)

Group 2: Cyclophosphamide group (CY; physiological saline with CY)

Group 3: Ginseng group (GIN; ginseng 100 mg/kg BW with CY)

Group 4: Levamisole group (LEV; levamisole 40 mg/kg BW with CY)

Group 5: PEG6000 group (PEG; PEG6000 100 mg/kg BW with CY)

Groups 6–9: OM-PEG (groups with OM-PEG at different doses of 50, 100, 150, and 200 mg/kg BW with CY, respectively).

**Figure 1:**
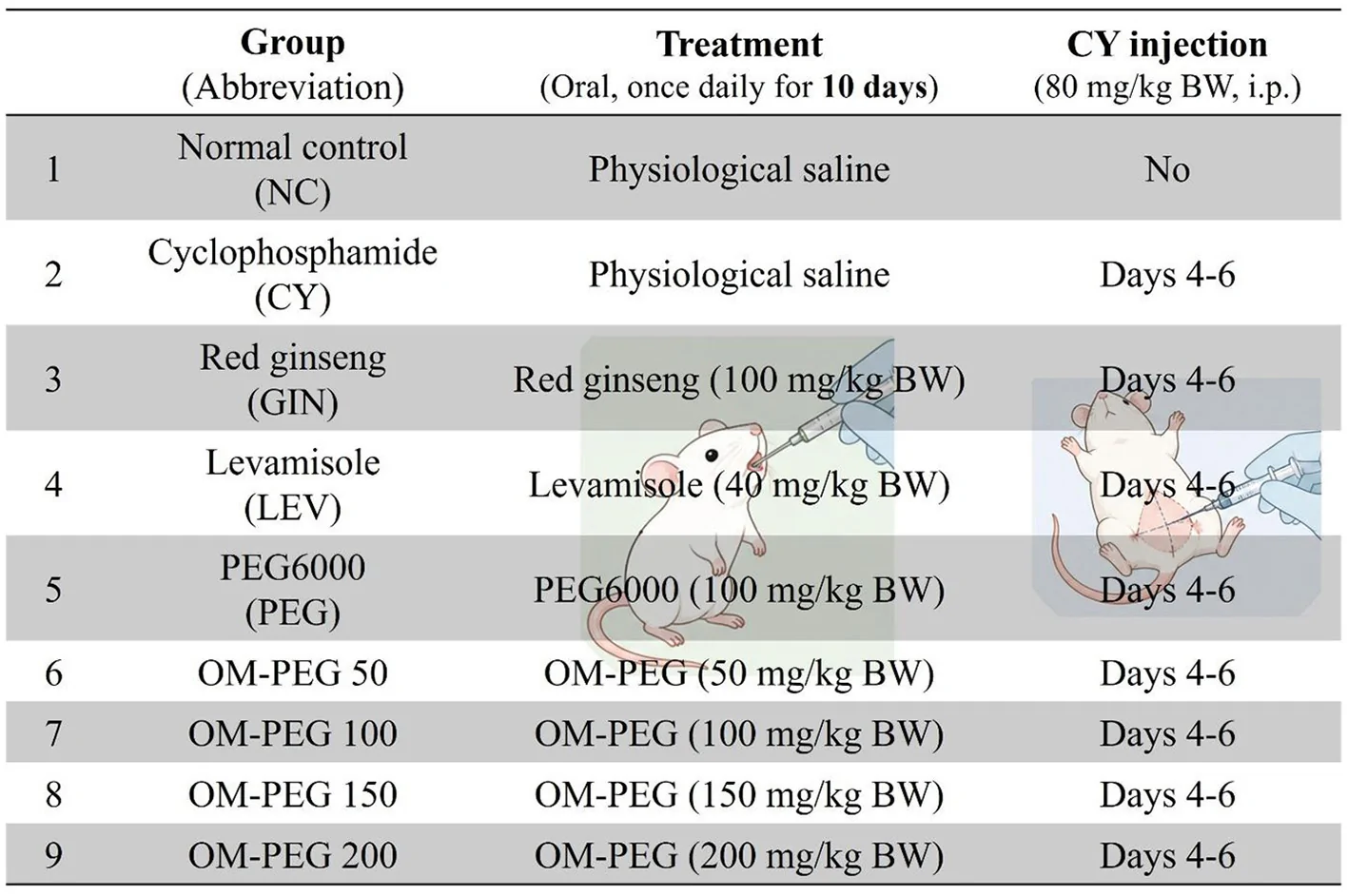
Experimental design.

The mice were orally administered one of the following treatments once daily for 10 consecutive days: saline, ginseng, levamisole, PEG6000, or OM-PEG. CY (Sigma-Aldrich, USA) was injected intraperitoneally at a dose of 80 mg/kg on days 4–6 to induce immunosuppression in all groups, except for the NC group. Ginseng and levamisole served as positive controls, CY served as the negative control, and PEG served as the vehicle (model) control. Following the final treatment, the animals were fasted for 24 h and then euthanized by gradual fill CO_2_ inhalation at a flow rate of 20%−30% of chamber volume per minute, followed by cervical dislocation. Samples were subsequently collected for further analysis (Figure 1).

### 2.5 Measurement of spleen index

The spleens from each group were harvested immediately after euthanasia. The spleens were weighed and rinsed with normal saline. The spleen indices were calculated using the following formula:

$$\begin{array}{l} \text{Spleen index }\left(\text{mg}/\text{g}\right)\;=\;\frac{\text{spleen weight }\left(\text{mg}\right)}{\text{ body weight before euthanasia }\left(\text{g}\right)} \end{array}$$

### 2.6 Preparation of peritoneal macrophages and splenocytes

Peritoneal macrophages were collected by intraperitoneal (i.p.) injection of 5 ml of ice-cold phosphate-buffered saline (PBS) supplemented with 3% FBS into the peritoneal cavity of each mouse. The cell suspension was subsequently collected and centrifuged, and the resulting cell pellets were resuspended in RPMI-1640 medium containing 10% fetal bovine serum (FBS), 1% penicillin-streptomycin, with the cell concentration adjusted to 1 × 10^6^ cells/mL for subsequent experiments.

Briefly, the harvested spleens of BALB/c mice were weighed and gently disrupted in ice-collected PBS to obtain a cell suspension. The resulting cell suspension was treated with 1 × RBC Lysis Buffer (eBioscience, USA) to remove red blood cells. The remaining cells were centrifuged at 400 × *g* for 10 min, and washed with PBS, and resuspended in RPMI-1640 medium. The cell density was adjusted to 2 × 10^6^ cells/mL for subsequent experiments.

### 2.7 Assay of nitric oxide (NO) production

Peritoneal macrophages were seeded at a density of 1 × 10^5^ cells/well in 96-well plate and incubated at 37 °C in a humidified incubator containing 5% CO_2_ for 1 h. Lipopolysaccharides (LPS, 1 μg/ml) was then added and cells were incubated for additional 24 h. Subsequently, supernatants (100 μl) were collected and reacted with Griess reagent (Promega, USA), and incubated at room temperature for 10 min. The absorbance (A) at 540 nm was measured to determine the nitrite levels in the macrophage culture supernatants.


$$\begin{array}{l} \text{NO production }\left(\text{\%}\right)\;=\;\frac{{\text{A}}_{540\text{ nm}}\;of\;the\;test\;group}{{\text{A}}_{540\text{ nm}}\;of\;the\;control\;group}\;\times \;100 \end{array}$$

### 2.8 Analysis of phagocytosis of peritoneal macrophages

Peritoneal macrophages were seeded at a density of 1 x 10^5^ cells/well in 96-well plate and incubated at 37 °C in a humidified incubator containing 5% CO_2_ for 24 h. After incubation, the cells were washed with PBS and then treated with 200 μl of 0.09% neutral red solution per well, followed by incubation at 37 °C for 30 min. After incubation, cells were washed with PBS and incubated with 200 μl of 0.09% neutral red solution per well at 37 °C for 30 min. The cells were then washed again with PBS, and 100 μl of a destaining solution containing 50% ethanol and 1% glacial acetic acid was added to each well. Absorbance was measured at 540 nm using a microplate reader (BioTek Instruments, USA).

### 2.9 Assay of splenic lymphocyte proliferation

Splenic lymphocytes (2 × 10^6^ cells/mL) were seeded into 96-well plates at a volume of 100 μl per well. Con A (5 μg/ml) and LPS (1 μg/ml) were added to the wells containing splenocytes and cultured at 37 °C for in a humidified atmosphere containing 5% CO_2_ 48 h, with RPMI medium serving as the control. Following the incubation, 25 μl of water-soluble tetrazolium salt (WST) from the EZ-Cytox Cell Viability Assay Kit (DaeilLab Service, Korea) was added to each well and incubated for 1 h. Absorbance was measured at 450 nm using a microplate reader.

### 2.10 Analysis of NK cell-mediated cytotoxicity assay of splenocytes

Splenocytes were co-cultured with YAC-1 cells as target cells at an effector-to-target ratio of 50:1 and incubated at 37 °C in a humidified atmosphere containing 5% CO_2_ for 24 h. NK cell activity was measured using the CytoTox 96^®^ Non-Radioactive Cytotoxicity Assay (Promega, USA), absorbance was measured at 490 nm, and the percentage of NK cell cytotoxicity was calculated according to the manufacturer's instructions.

### 2.11 Quantitative RT-PCR (qRT-PCR) analysis

Total RNA was extracted from LPS-stimulated peritoneal macrophages and ConA- and LPS-stimulated splenic lymphocytes using TRI Reagent^®^ (Molecular Research Center, Inc., Cincinnati, USA). The total RNA was dissolved in nuclease-free water, its concentration was measured using a nanophotometer (Implen, Germany), and cDNA was synthesized using a High-Capacity cDNA Reverse Transcription Kit (Applied Biosystems, USA). The cDNA from these samples was mixed with TB Green^®^ Premix Ex Taq™ II (Takara Bio Inc., Japan) and specific primers (Table 1), with β*-actin* used as an internal reference gene, and immune gene expression was analyzed using the QuantStudio™ 3 Flex Real-Time PCR System (Applied Biosystems, USA), and relative gene expression levels were calculated using the 2–ΔΔCt method.

**Table 1:** Primers used for qPCR analysis of specific genes in mice.

Gene	Sequence of primer (5'−3')	
	Forward primer	Reverse primer
*IL-1β*	GGGCCTCAAAGGAAAGAATC	TACCAGTTGGGGAACTCTGC
*IL-2*	CCTGAGCAGGATGGAGAATTACA	TCCAGAACATGCCGCAGAG
*IL-4*	ACAGGAGAAGGGACGCCAT	GAAGCCCTACAGACGAGCTCA
*IL-6*	AGTTGCCTTCTTGGGACTGA	CAGAATTGCCATTGCACAAC
*IFN-γ*	CTCAAGTGGCATAGATGT	GAGATAATCTGGCTCTGCAGGATT
*TNF-α*	ATGAGCACAGAAAGCATGATC	TACAGGCTTGTCACTCGAATT
*iNOS*	TTCCAGAATCCCTGGACAAG	TGGTCAAACTCTTGGGGTTC
*COX-2*	AGAAGGAAATGGCTGCAGAA	GCTCGGCTTCCAGTATTGAG
*β-actin*	CCACAGCTGAGAGGGAAATC	AAGGAAGGCTGGAAAAGAGC

### 2.12 Statistical analysis

The differences between groups were assessed using one-way analysis of variance (ANOVA), followed by the Duncan *post hoc* test, with data analyzed using IBM SPSS Statistics software (Ver. 23, IBM, USA). A *p*-value of less than 0.05 was considered indicative of a significant difference between treatment groups.

## 3 Results

### 3.1 Identification of fatty acids in OM-PEG

**Figure 2:**
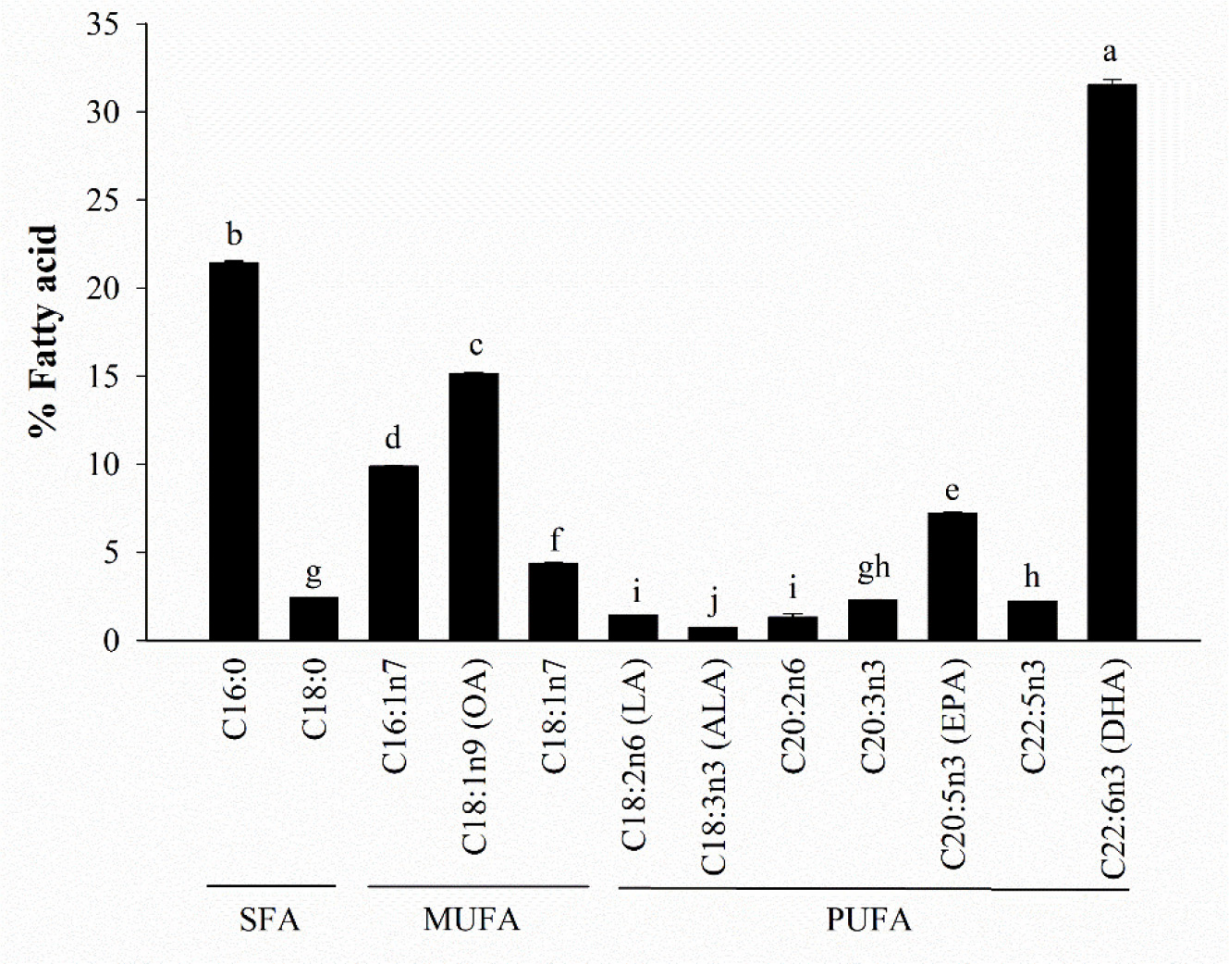
The composition of fatty acid profile in OM-PEG. Values are presented as means ± SD (*n* = 5). The letters (a-j) refer to significant differences (*p* < 0.05) in the amounts of fatty acids.

To determine the fatty acid composition of OM-PEG, gas chromatography analysis was performed (Figure 2). A total of 12 fatty acids were identified, comprising two saturated fatty acids (SFAs; C16:0 and C18:0), three monounsaturated fatty acids (MUFAs; C16:1n7, C18:1n9, and C18:1n7), and seven polyunsaturated fatty acids (PUFAs; C18:2n6, C18:3n3, C20:2n6, C20:3n3, C20:5n3, C22:5n3, and C22:6n3). PUFAs constituted the highest proportion of total fatty acids in OMPEG (46.71 ± 0.20%), followed by MUFAs (29.43 ± 0.09%) and SFAs (23.86 ± 0.11%).

### 3.2 Effect of OM-PEG on spleen index

**Figure 3:**
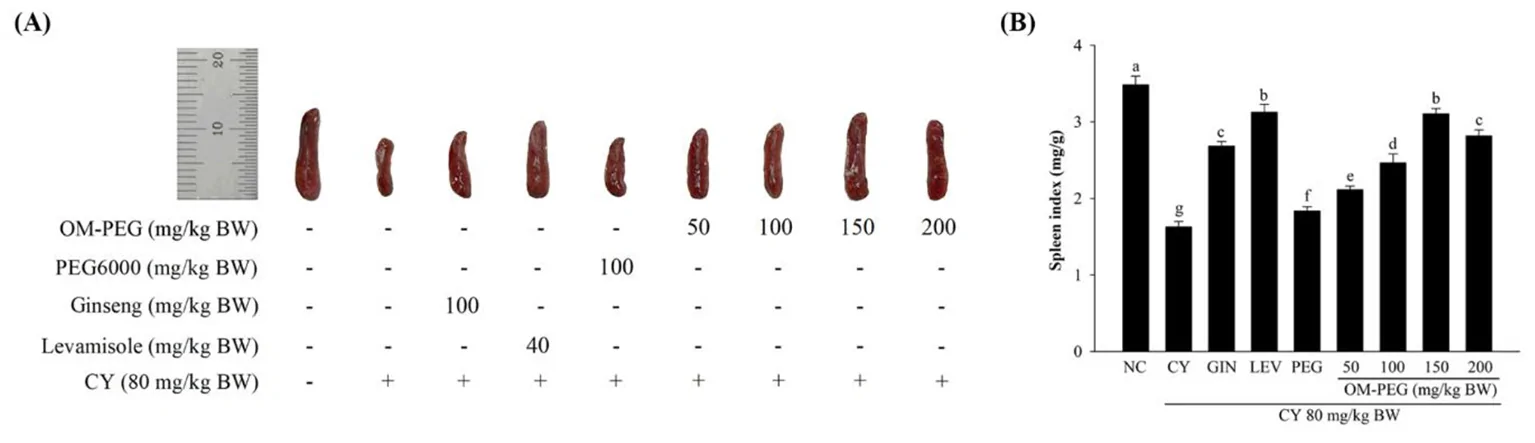
Effect of OM-PEG on spleen in immunosuppressed mice. **(A)** Representative pictures of spleen size and **(B)** spleen index. Values are presented as mean ± SD (*n* = 3). Data were statistically analyzed using one-way analysis of variance (ANOVA), followed by Duncan's *post hoc* test. Different letters indicate statistically significant differences at *p* < 0.05 among treatment groups. NC: Normal control group; CY: CY-treated group; GIN: CY + ginseng (100 mg/kg BW); LEV: CY + levamisole (40 mg/kg BW); PEG: CY + PEG6000 (100 mg/kg BW). OM-PEG: CY + OM-PEG (50–200 mg/kg BW).

The spleen is one of the organs closely associated with immune function in humans and mice. As shown in Figure 3, the spleen size and index in the NC group were significantly higher (*p* < 0.05) than those in the other groups injected with CY, indicating that CY impaired the immune system of the mice. Compared to the CY-treated mice, the OM-PEG group exhibited a significant increase in spleen indices, with the 150 mg/kg BW OM-PEG group reaching 3.10 ± 0.07 mg/g, the highest value among the OM-PEG groups. Additionally, the positive groups (GIN and LEV) showed spleen indices of 2.68 ± 0.06 and 3.13 ± 0.10 mg/g, respectively, whereas the PEG model group had a spleen index of 1.84 ± 0.05 mg/g. The OM-PEG groups at 150 and 200 mg/kg BW exhibited spleen indices comparable to those of the positive control groups. Therefore, the results indicated that OM-PEG promoted the recovery of both spleen size and spleen index in CY-induced immunosuppressed mice.

### 3.3 Effect of OM-PEG on splenic lymphocyte proliferation

**Figure 4:**
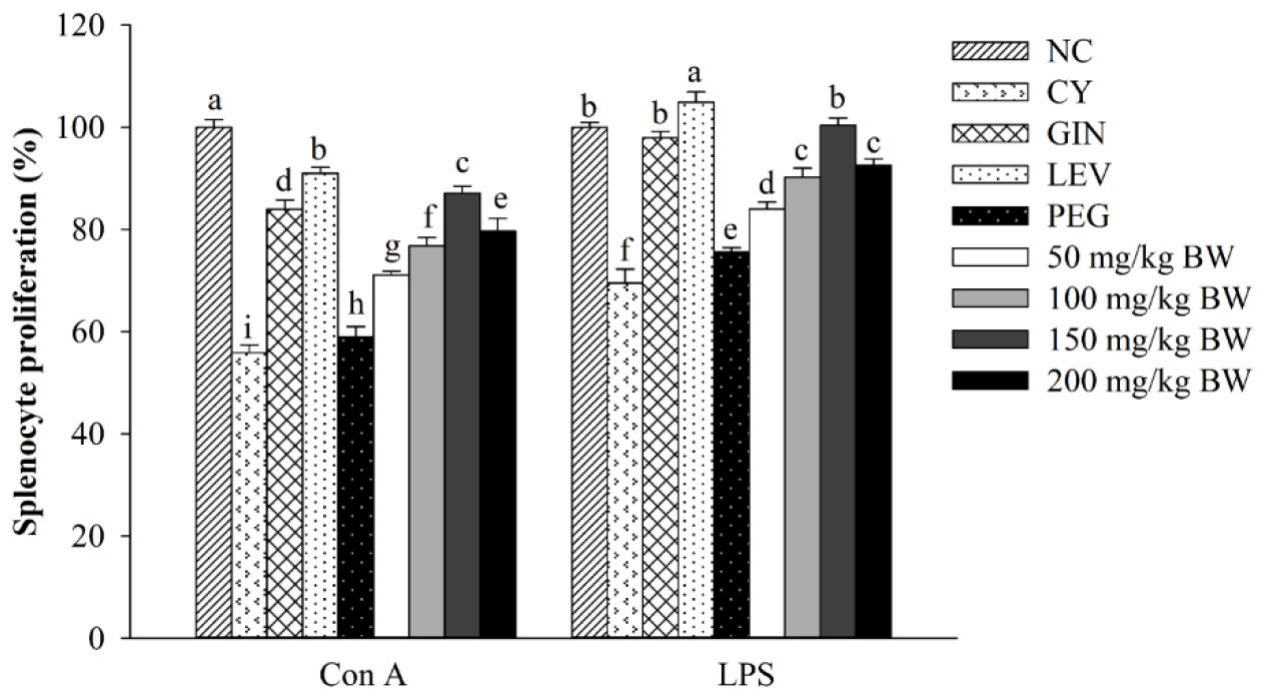
OM-PEG increased mitogen-stimulated splenocyte proliferation. Values are presented as mean ± SD (*n* = 3). Data were statistically analyzed using one-way analysis of variance (ANOVA), followed by Duncan's *post hoc* test. Different letters indicate statistically significant differences at *p* < 0.05 among treatment groups. NC, normal control group; CY, CY-treated group; GIN, CY + ginseng (100 mg/kg BW); LEV, CY + levamisole (40 mg/kg BW); PEG: CY + PEG6000 (100 mg/kg BW). OM-PEG: CY + OM-PEG (50–200 mg/kg BW).

Figure 4 shows that OM-PEG stimulated the proliferation of spleen lymphocytes following mitogen stimulation. Accordingly, a significant reduction in lymphocyte proliferation following both Con A and LPS stimulation was observed in CY-treated mice (*p* < 0.05) compared to the NC-treated mice. Upon stimulation with Con A or LPS, OM-PEG treatment in combination with CY enhanced the proliferation of splenocytes in a dose-dependent manner at 50–150 mg/kg BW, whereas the higher concentration of 200 mg/kg BW slightly reduced the effect.

### 3.4 Effect of OM-PEG on NK cell activity of splenocytes

**Figure 5:**
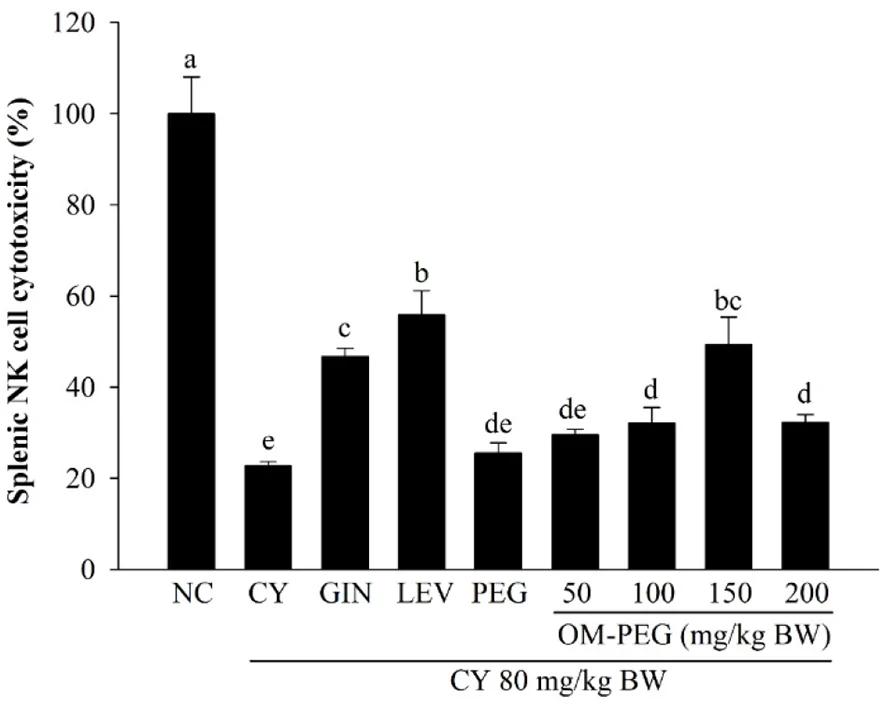
OM-PEG increased splenic NK cell cytotoxic activity. Values are presented as mean ± SD (*n* = 3), and statistical differences were evaluated using one-way analysis of variance (ANOVA) followed by Duncan's *post hoc* test. Different letters indicate statistically significant differences at *p* < 0.05 among treatment groups. NC: Normal control group; CY: CY-treated group; GIN: CY + ginseng (100 mg/kg BW); LEV: CY + levamisole (40 mg/kg BW); PEG: CY + PEG6000 (100 mg/kg BW). OM-PEG: CY + OM-PEG (50–200 mg/kg BW).

NK cells are a major population of cytotoxic lymphocytes, and research on NK cells is being conducted across multiple fields of immunology, including tumor immunology and infectious diseases (27). Splenocyte cytotoxicity was assessed against NK-sensitive tumor cells (YAC-1). As shown in Figure 5, NK cell activity was significantly decreased following intraperitoneal injection of CY. OM-PEG progressively enhanced NK cells activity in splenocytes. Notably, splenic NK cell activity was restored to normal levels when OM-PEG was administered at a dose of 150 mg/kg BW. Additionally, OM-PEG enhanced NK cell activity in splenocytes in a dose-dependent manner.

### 3.5 Effect of OM-PEG on immune-related gene expression in splenic lymphocytes

**Figure 6:**
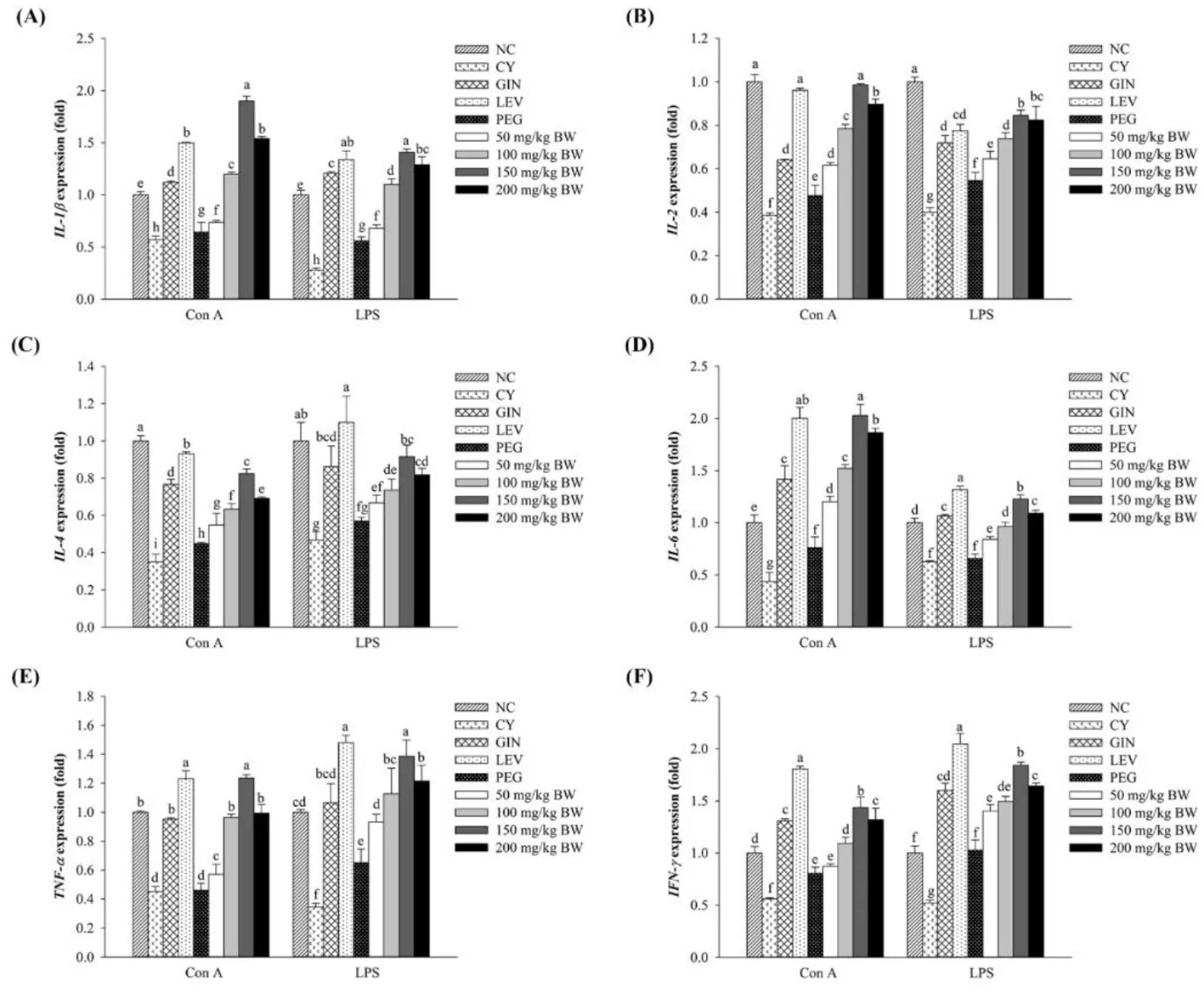
Effects of OM-PEG on mRNA expression of immune genes in mitogen-stimulated splenic lymphocytes. The mRNA levels of *IL-1*β **(A)**, *IL-2* **(B)**, *IL-4* **(C)**, *IL-6* **(D)**, *TNF-*α **(E)**, and *IFN-*γ **(F)**. Data are expressed as mean ± SD (*n* = 3), and differences among treatment groups were analyzed using one-way analysis of variance (ANOVA) followed by Duncan's *post hoc* test. Different letters indicate statistically significant differences at *p* < 0.05 among treatment groups. NC, normal control group; CY, CY-treated group; GIN, CY + ginseng (100 mg/kg BW); LEV, CY + levamisole (40 mg/kg BW); PEG, CY + PEG6000 (100 mg/kg BW). OM-PEG, CY + OM-PEG (50–200 mg/kg BW).

The effects of OM-PEG on the expression of immune-related genes in the mice were investigated (Figure 6). Compared to NC-treated mice, the intraperitoneal injection of CY significantly decreased the relative mRNA expression levels of *IL-1*β, *IL-2, IL-4, IL-6, TNF-*α, and *IFN-*γ. However, treatment with OM-PEG significantly enhanced the expression of immune-related genes (*IL-1*β, *IL-2, IL-4, IL-6, TNF-*α, and *IFN-*γ) in splenic cells following stimulation with T-cell and B-cell mitogens, particularly at doses of 50–150 mg/kg BW. Notably, the expression levels of these genes were lower at a dose of 200 mg/kg BW of OM-PEG compared to the 150 mg/kg BW dose.

### 3.6 Effect of OM-PEG on phagocytic activity of peritoneal macrophages

**Figure 7:**
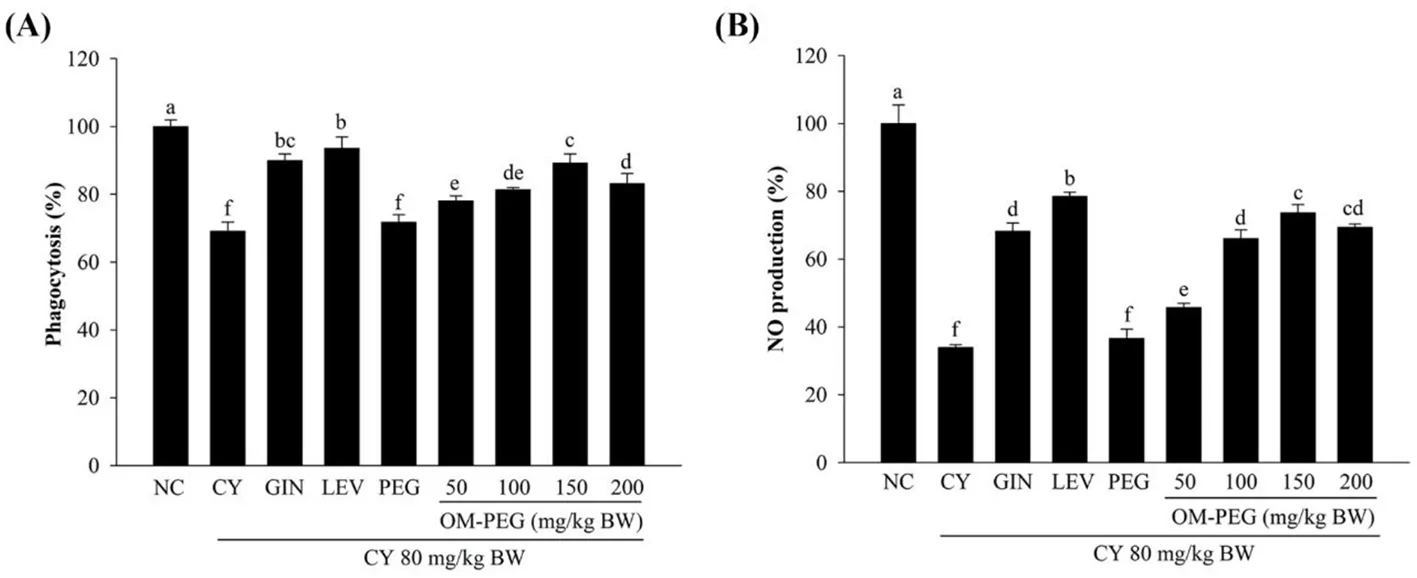
Effects of OM-PEG on peritoneal macrophages. **(A)** Macrophage phagocytosis. **(B)** NO production. Results are presented as mean ± SD (*n* = 3), with statistical differences among treatment groups assessed using one-way analysis of variance (ANOVA) followed by Duncan's *post hoc* test. Different letters indicate statistically significant differences at *p* < 0.05 among treatment groups. NC, Normal control group; CY, CY-treated group; GIN, CY + ginseng (100 mg/kg BW); LEV, CY + levamisole (40 mg/kg BW); PEG, CY + PEG6000 (100 mg/kg BW). OM-PEG: CY + OM-PEG (50–200 mg/kg BW).

Macrophages, including phagocytes, play a crucial role in regulating innate immune responses by engulfing and eliminating microorganisms or cancer cells through phagocytosis (28). The phagocytic function of macrophages is the one of the most important non-specific immune responses against foreign organisms. The phagocytic activity of primary peritoneal macrophages from CY-induced immunosuppressed mice (Figure 7A), was measured by the Neutral Red uptake method. The mice in the CY and PEG groups showed similar phagocytic activity, with both group exhibiting greater reduction compared to the NC-treated mice. Oral administration of OM-PEG significantly enhanced the phagocytosis of primary peritoneal macrophages at doses of 50-150 mg/kg BW, exhibiting a dose-dependent increase. Compared with the GIN and LEV groups, OM-PEG treatment in CY-induced immunosuppressed mice restored phagocytic activity to similar levels at doses 150 and 200 mg/kg BW.

### 3.7 Effect of OM-PEG on NO production of peritoneal macrophages

Nitric oxide (NO) production by macrophage cells is known to protect host cells from infection (29). As shown in Figure 7B, NO production of peritoneal macrophages was lowest in the CY-treated mice, and was comparable that in the PEG group, which served as the model control. On the other hand, OM-PEG treatment led to a dose-dependent increase in NO production at doses of 50–150 mg/kg BW, although this effect was slightly reduced at 200 mg/kg BW compared with that at 150 mg/kg BW.

### 3.8 Effect of OM-PEG on immune-related gene expression in peritoneal macrophages

**Figure 8:**
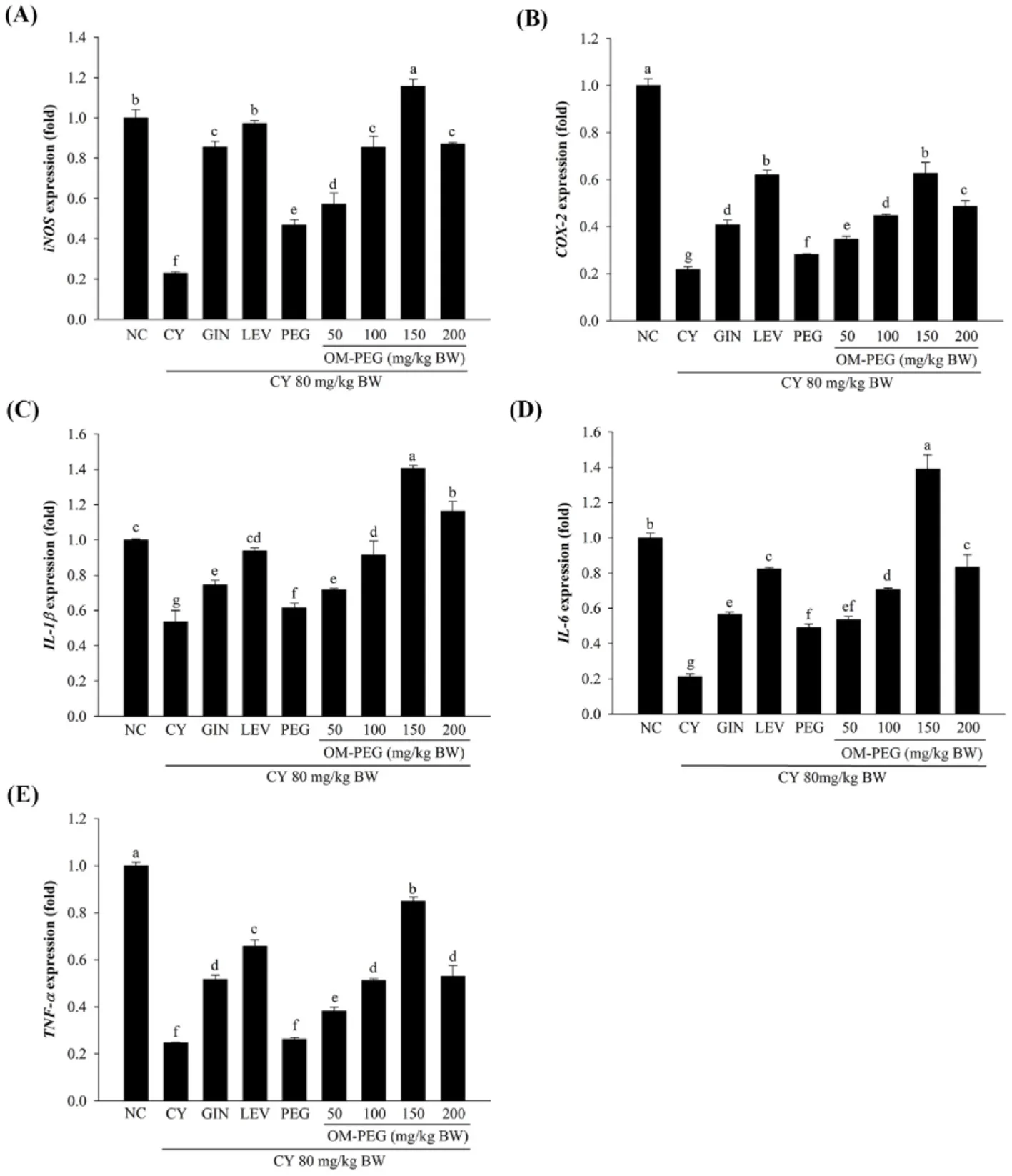
Effects of OM-PEG on immune-regulated gene expression in LPS-stimulated peritoneal macrophages. The mRNA levels of *iNOS* **(A)**, *COX-2* **(B)**, *IL-1*β **(C)**, *IL-6* **(D)**, and *TNF-*α **(E)**. Values are presented as mean ± SD (*n* = 3), and statistical differences among treatment groups were determined using one-way analysis of variance (ANOVA) followed by Duncan's *post hoc* test. Different letters indicate statistically significant differences at *p* < 0.05 among treatment groups. NC, normal control group; CY, CY-treated group; GIN, CY + ginseng (100 mg/kg BW); LEV, CY + levamisole (40 mg/kg BW); PEG, CY + PEG6000 (100 mg/kg BW). OM-PEG, CY + OM-PEG (50–200 mg/kg BW).

The data showed that CY group markedly downregulated the mRNA expression of *iNOS, COX-2, IL-1*β, *IL-6*, and *TNF-*α (Figure 8). Different doses of OM-PEG (50, 100, 150, and 200 mg/kg BW) were administered to CY-treated mice. The expression of these immune-associated genes increased in a dose-dependent manner, except at OM-PEG dose of 200 mg/kg BW which exhibited a slight reduction compared to 150 mg/kg BW. Notably, peritoneal macrophages treated with OM-PEG at a dose of 150 mg/kg BW had comparable or greater gene expression levels than with the GIN and LEV groups.

## 4 Discussion

Natural compounds derived from various marine organisms, including *Arctoscopus japonicus* eggs, *Nibea japonica* skin, *Strongylocentrotus nudus* eggs, and the body wall of *Apostichopus japonicus* (13, 30–32), have been shown to alleviate CY-induced immunosuppression in mice. *O. mykiss* is a globally cultured cold-water fish species known for its high nutritional value. Gwon et al. reported that egg lipids from *O. mykiss*, which contain high levels of PUFAs (51.92% of total fatty acids), alleviated inflammatory effects in macrophages. However, the immunomodulatory potential of *O. mykiss* lipids in CY-induced immunosuppressed mice has not yet been investigated.

OM-PEG was prepared by conjugating PEG 6,000 with lipids extracted from *O. mykiss* eggs. PEG, a non-toxic hydrophilic polymer, enhances the steric stability and integrity of lipid-based nanoparticles by forming a hydrated protective layer (33). Lipids from *A. japonicus* and *A. amurensis* eggs have previously demonstrated immunomodulatory effects in cyclophosphamide-induced immunosuppressed mice (13, 34). In the present study, GC-FID analysis indicated that lipids isolated from *O. mykiss* eggs are rich in PUFAs, including EPA (20:5n-3) and DHA (22:6n-3), which have been widely reported to possess immunomodulatory and anti-inflammatory properties (35). Bioactive EPA and DHA regulate innate and adaptive immune responses by modulating membrane phospholipid composition, inflammatory eicosanoid synthesis, cytokine production, immune cell signaling, and the biosynthesis of specialized pro-resolving mediators (SPMs), including EPA-derived resolvins and DHA-derived docosanoids (36–38). Omega-3 PUFAs also regulates macrophage activation and cytokine production, thereby contributing to the maintenance of immune homeostasis (39). Therefore, the immune-enhancing effects observed in the present study may be attributed at least in part to the high PUFA content of OM-PEG. Furthermore, PEGylation may enhance the stability and dispersion of the lipid formulation, thereby improving the delivery and bioavailability of its bioactive lipid components. Consequently, the immunomodulatory activity of OM-PEG may result from the combined effects of its bioactive lipids and the stabilizing properties of PEGylation. Additionally, other lipid-soluble nutrients naturally present in fish eggs, such as vitamin E, may contribute to the immunomodulatory effects of OM-PEG. Vitamin E is the principal lipid-soluble antioxidant that protects highly unsaturated PUFAs, including EPA and DHA, from lipid peroxidation, thereby preserving their biological activity and membrane function (40). Previous studies have shown that vitamin E can influence immune function by enhancing T-cell-mediated responses, supporting lymphocyte proliferation, and regulating inflammatory processes (41). Therefore, although vitamin E content was not determined in the present study, its potential involvement in the immune-restorative effects observed following OM-PEG administration cannot be ruled out.

The spleen is a major secondary lymphoid organ composed of immune cells such as monocytes, macrophages, and B and T lymphocytes, which collectively mediate immunological responses. Spleen-related parameters, such as spleen index and NK cell activity, positively correlate with the function of innate immunity in BALB/c mice (7, 42, 43). In this study, the spleen index was markedly reduced in the CY-treated group indicating that CY treatment stressed the mice and impaired their immune function. Administration of OM-PEG significantly restored the spleen index in the CY-treated mice, with the greatest improvement observed at 150 mg/kg BW. A similar improvement in the spleen index has been reported following treatment with erucic acid, a monounsaturated omega-fatty acid (44), suggesting that fatty acids may help to mitigate chemically induced immune dysfunction and further support the immunomodulatory potential of naturally derived bioactive compounds. Although the 200 mg/kg BW group also improved the spleen index compared with the CY group; however, the effect was lower than that observed at 150 mg/kg BW. These findings suggest that 150 mg/kg BW produced the greatest improvement in spleen recovery under the conditions of the present study, whereas increasing the dose to 200 mg/kg BW did not provide any additional benefits.

Lymphocytes are a major class of white blood cells comprising T lymphocytes and B lymphocytes, both of which play an important role in immune responses by defending against pathogens, diseases, and infections (45, 46). Lymphocyte proliferation is a key indicator of the host's immune function and reflects the activation of T and B lymphocytes in response to antigens or mitogens (47). Con A and LPS are commonly used mitogens that selectively stimulate T and B cells, respectively (48). In the present study, CY treatment markedly suppressed the proliferative responses of splenic lymphocytes to both Con A and LPS, indicating impaired T- and B-cell function. However, treatment with OM-PEG (50–200 mg/kg BW) significantly enhanced both Con A- and LPS-induced splenic lymphocyte proliferation in CY-treated mice. The greatest enhancement in splenic lymphocyte proliferation was observed at 150 mg/kg BW. A slight reduction was observed at 200 mg/kg BW; however, the response remained significantly greater than that in the CY-treated group. In agreement with these findings, a previous study showed that DHA improved splenocyte immune competence in an immunosuppressive mouse model (49), supporting the potential contribution of omega-3 PUFAs to this immunomodulatory activity of OM-PEG. The enhanced response to both Con A and LPS suggests that OM-PEG may improve the responsiveness of both T-cell- and B-cell-associated immune responses. In addition to T- and B-cells, NK cells may contribute to adaptive immunity (50). NK cells play a pivotal role in nonspecific antiviral and antitumor defense and also regulate the adaptive immune system through secretion of cytokines (51, 52). In the present study, treatment with OM-PEG significantly enhanced splenic NK cell activity in CY-induced immunosuppressed mice. The highest NK cell activity was observed at 150 mg/kg BW with slight reduction at 200 mg/kg BW, but the activity remained significantly higher than that of the CY group. Furthermore, at 150 mg/kg BW, NK cell activity was similar to that of the positive group (levamisole and ginseng). These findings are consistent with previous reports showing that sea buckthorn pulp oil, which is rich in unsaturated fatty acids, phytosterols, and vitamins A and E, enhances NK cell cytotoxicity in cyclophosphamide-induced immunosuppressed mice (53). The comparable effects observed in these studies suggest that bioactive lipids and other lipid-soluble compounds may play a role in restoring NK cell function. Collectively, these findings further support the immunomodulatory potential of OM-PEG.

Macrophages are major components of the innate system and, plays a pivotal role in host defense, engulfing and eliminating pathogens, microorganisms, or cancer cells through phagocytosis (28). Upon activation, macrophages express iNOS, which synthesizes a significant amount of NO, an important antimicrobial and antitumor effector molecule that helps prevent pathogen invasion (54). In addition to phagocytic activity, macrophages regulate immune homeostasis by functioning as both regulatory and effector cells, thereby linking innate and adaptive immune responses against pathogens (55). In the current study, cyclophosphamide (CY) treatment significantly reduced phagocytic activity and NO production of peritoneal macrophages, indicating impaired immune function. In contrast, treatment with OM-PEG significantly increased phagocytic activity and NO production in CY-treated mice, suggesting that OM-PEG can effectively restore macrophage function in CY-induced immune-suppressed mice. These findings are consistent with previous reports showing that *A. japonicus* lipids rich in PUFAs significantly enhanced phagocytosis and NO production in cyclophosphamide-induced immunosuppressed mice (13). Therefore, these findings suggest that OM-PEG alleviates CY-induced immune dysfunction by enhancing macrophage function. Cytokines are small signaling proteins that play a crucial role in immune regulation. They are produced by various cell types, including immune cells such as macrophages, T cells, and B cells, and regulate the growth, differentiation, and activity of immune cells (56). T helper (Th) 1 cells are associated with the production of cytokines such as IL-1β, IL-2, IFN-γ, and TNF-α, whereas Th2 cells are associated with the production of IL-4, IL-5, IL-6, and IL-10 (57). In the present study, OM-PEG significantly upregulated the expression of immune-related genes, including *IL-1*β, *IL-2, IL-4, IL-6, TNF-*α, and *IFN-*γ, in mitogen-stimulated splenic lymphocytes from CY-treated mice. Notably, OM-PEG administered at 150 mg/kg restored cytokine levels to values comparable or higher than those observed in the NC group, indicating its potential to restore the impaired immune response in CY-induced immunosuppression. In addition to splenic lymphocytes, activated macrophages produce cytokines such as IFN-γ, IL-1β, IL-6, IL-10, and TNF-α, which contribute to recruitment and activation of other immune cells for the initiation of the adaptive immune response (58). In the present study, to further evaluate the effect of OM-PEG on macrophage function and immune restoration, peritoneal macrophages were isolated and stimulated with LPS to assess the expression of macrophage-associated immune genes. CY administration markedly reduced the LPS-induced mRNA expression of iNOS, COX-2, IL-1β, IL-6, and TNF-α in peritoneal macrophages. However, OM-PEG supplementation significantly restored the expression of these immune-associated genes in a dose-dependent manner. Likewise, our previous study demonstrated that PUFA-rich lipids from Asterias amurensis skin induced the expression of immune-related genes in both mitogen-stimulated splenic lymphocytes and peritoneal macrophages from CY-treated mice (34). OM-PEG at 150 mg/kg BW exhibited comparable or even stronger effects than the GIN and LEV groups, indicating improved restoration of macrophage responsiveness. Although OM-PEG at 200 mg/kg BW significantly restored the expression of immune-related genes, its effects were slightly lower than those observed at 150 mg/kg BW, which may reflect a plateau in the immunomodulatory response at higher doses. Taken together, these findings suggest that OM-PEG has immunomodulatory effects by enhancing immune response in both splenic lymphocytes and peritoneal macrophages in CY-induced immunosuppressed mice.

## Strengths and limitations of the study

The major strength of this study is its comprehensive evaluation of the immunomodulatory effects of OM-PEG in CY-induced immunosuppressed mice using multiple immune function-related parameters, including spleen index, splenic lymphocyte proliferation, NK cell activity, peritoneal macrophage phagocytosis, nitric oxide production, and the expression of immune-related genes in splenic lymphocytes and peritoneal macrophages. Consistent improvements were observed across all parameters, suggesting that OM-PEG may contribute to the restoration of immune function under immunosuppressive conditions. However, this study has several limitations that should be acknowledged. First, the relatively small number of animals (*n* = 3 per group) may limit the statistical power and generalizability of the findings. Therefore, future studies using larger experimental groups are necessary to confirm the immunomodulatory efficacy of OM-PEG. Second, although immune parameters were extensively evaluated, food intake and changes in body weight during the experimental period were not monitored. Third, although the fatty acid composition of OM-PEG was determined, other nutritional components such as vitamin E and mineral contents were not assessed. In particular, vitamin E may influence immune regulation; therefore, future studies incorporating a complete nutritional profile of OM-PEG are required to further validate these findings.

In conclusion, this study demonstrated that OM-PEG effectively modulates immune responses in cyclophosphamide (CY)-induced immunosuppressed mice. OM-PEG enhanced immune function by improving the spleen index, stimulating splenic lymphocyte proliferation, and increasing NK cell activity. Furthermore, OM-PEG enhanced NO production and phagocytic activity in peritoneal macrophages. Additionally, OM-PEG significantly modulated the expression of immune-related genes in both peritoneal macrophages and splenic lymphocytes. Overall, these findings suggest that OM-PEG exhibits immunomodulatory effects and alleviates cyclophosphamide-induced immunosuppression in mice, highlighting its potential as a natural marine-derived functional food ingredient for supporting immune health. Further studies are warranted to elucidate the underlying molecular mechanisms and determine the contributions of individual fatty acids to the immunomodulatory effects of OM-PEG.

## Data Availability

The raw data supporting the conclusions of this article will be made available by the authors, without undue reservation.
